# Lenacapavir in the Treatment and Prevention of HIV-1 Infection: A Narrative Review of Clinical Efficacy and Resistance Selection Risk

**DOI:** 10.7759/cureus.119393

**Published:** 2026-11-09

**Authors:** Umut Tasbas

**Affiliations:** 1 General Practice, Dr. Lütfi Kırdar Kartal Eğitim ve Araştırma Hastanesi, Istanbul, TUR

**Keywords:** "anti-hiv agents", capsid, hiv aids, hiv drug resistance, lenacapavir

## Abstract

Lenacapavir is a first-in-class HIV-1 capsid inhibitor. By acting on a structural protein rather than a viral enzyme, it inhibits several stages of the replication cycle and shows no cross-resistance with the available drug classes; a half-life of 8-12 weeks after subcutaneous administration allows a six-monthly regimen. A narrative review was conducted, searching PubMed and ClinicalTrials.gov up to July 2026 and drawing on phase 2 and phase 3 trials, structural and modelling studies, and regulatory documents. In virologically suppressed adults undergoing a planned regimen switch, the week-48 treatment differences for bictegravir/lenacapavir (ARTISTRY-1 and ARTISTRY-2) and for weekly oral islatravir/lenacapavir (ISLEND-1 and ISLEND-2) ranged from −1.0 to 0.3 percentage points, with the upper bound of each 95% confidence interval lying below the prespecified non-inferiority margin of 4 percentage points. These are investigational switch regimens: lenacapavir itself is currently approved only for multidrug-resistant HIV-1 infection and for pre-exposure prophylaxis, whereas neither the bictegravir/lenacapavir nor the islatravir/lenacapavir combination has yet been authorised. In multidrug-resistant infection, 85% of participants in CAPELLA had HIV-1 RNA < 50 copies/mL at week 156 when missing data were excluded. In prevention, no infection occurred among the 2,134 participants in the lenacapavir arm of PURPOSE 1 (0.00/100 person-years; incidence rate ratio vs background: 0.00, 95% CI: 0.00-0.04), whereas the incidence in the emtricitabine-tenofovir alafenamide arm did not differ from the background incidence (incidence rate ratio: 0.84, 95% CI: 0.55-1.28). The pharmacokinetic property that gives lenacapavir its strength is argued to be, at the same time, its principal vulnerability. As a pharmacological concern, the subtherapeutic concentration tail can create a state of functional monotherapy in which resistance may be selected when the virus replicates at concentrations that suppress the wild-type but not a resistant variant (the mutant selection window). Modelling predicts de novo resistance 106-235 days after the last injection and, for N74D and the double mutants, a selection window overlapping even the steady-state concentrations of the six-monthly regimen, so that these mutants may be selected not only during the post-discontinuation tail but also while dosing continues as planned. Resistance actually observed in clinical studies has been limited and conditional: N74D arose in the two participants infected in the lenacapavir arm of PURPOSE 2 despite expected drug concentrations, and in CAPELLA every participant who developed resistance had a background regimen that was inactive or inadequately taken, indicating that the vulnerability is manageable. Lenacapavir therefore requires careful patient selection, fully active companion drugs, and a planned discontinuation strategy.

## Introduction and background

According to World Health Organization data for the end of 2025, an estimated 41 million people are living with HIV worldwide, and 570,000 deaths from HIV-associated causes were reported in 2025 [1]. The 2030 targets of the Joint United Nations Programme on HIV/AIDS envisage providing treatment to 40 million people and antiretroviral-based prevention to 20 million [2].

Against this background, lenacapavir introduces innovation on two complementary levels. The first is the molecular target. The capsid is the cone-shaped protein shell that encloses the viral genome; lenacapavir is the first capsid inhibitor used in the treatment of HIV, and, rather than blocking a viral enzyme as the existing drug classes do, it acts on this structural protein, simultaneously inhibiting steps that range from transport of the capsid to the nucleus through to maturation of the virion [3,4]. Because this target is distinct from those of the available classes, viruses that are resistant to them remain susceptible to lenacapavir, an absence of cross-resistance that makes it particularly valuable in patients whose therapeutic options are exhausted [4]. The second level is pharmacokinetic: the prolonged elimination half-life makes a six-monthly regimen feasible and removes the need for daily dosing [5].

The molecule obtained approval in the European Union in August 2022 and in the United States in December of the same year, for multidrug-resistant infection in which a suppressive regimen could not otherwise be constructed [5,6]. The pre-exposure prophylaxis (PrEP) indication was approved in June 2025 by the Food and Drug Administration (FDA) and, in August 2025, by the European Medicines Agency (EMA) [7]. These two settings are clinically distinct: in treatment, lenacapavir is combined with other fully active antiretrovirals to suppress established infection, whereas, in prevention, it is given alone to HIV-negative individuals to prevent acquisition.

This distinction bears directly on resistance. Lenacapavir has a comparatively low genetic barrier, in that a single capsid substitution can reduce susceptibility, and its prolonged pharmacokinetic tail (the slowly declining drug concentrations that persist for months after the last dose) can leave the virus exposed to subtherapeutic levels. How far these properties translate into a clinically meaningful risk of resistance selection, especially where lenacapavir acts without the protection of companion drugs, has not yet been systematically appraised. The aim of this review is therefore to synthesise the available evidence on the clinical efficacy of lenacapavir in the treatment and prevention of HIV-1 infection, with particular attention to the risk of resistance selection. Its objectives are to examine the drug's mechanism of action and pharmacokinetics, to appraise the efficacy evidence in treatment and in prevention, and to analyse the determinants of resistance and their clinical implications. It argues that the pharmacokinetic property that gives lenacapavir its strength is at the same time its principal vulnerability, in that it creates a window of subtherapeutic concentrations conducive to the selection of resistance; this risk is present not only after discontinuation but also while dosing continues as planned.

Review methodology

This is a narrative review. PubMed/MEDLINE and ClinicalTrials.gov were searched for records published up to July 2026, using the terms "lenacapavir", "GS-6207", "capsid inhibitor", "HIV-1", "pre-exposure prophylaxis", and "resistance", supplemented by the United States and European product information and the reference lists of retrieved articles. Records reporting original clinical, virological, structural, or pharmacokinetic data on lenacapavir, or its regulatory information, were eligible; phase 2 and phase 3 trials, structural and pharmacokinetic or pharmacodynamic modelling studies, and resistance analyses were prioritised; conference and manufacturer sources were used only where peer-reviewed data were unavailable; and records not concerning lenacapavir were excluded. After title, abstract, and full-text screening, the sources were selected by the author, who extracted the data from the primary publications and synthesised them narratively by theme. As a narrative review, it involved no formal protocol, PRISMA flow diagram, or structured risk-of-bias assessment, and no meta-analysis was performed; where available, effect estimates are reported with 95% confidence intervals.

Because the included studies differ in design, population, and duration of follow-up, their results are compared descriptively rather than pooled, and direct numerical comparisons between trials should be interpreted with caution.

## Review

Pharmacology and mechanisms of action

The absorption of lenacapavir differs markedly according to the route. With oral administration, maximum plasma concentration is reached in about four hours, and the half-life is 10-12 days; with subcutaneous administration, peak plasma concentrations are reached 84 days after dosing, and the half-life is 8-12 weeks [5]. Absolute oral bioavailability lies between 6% and 10%, which explains the high oral doses used in the loading phase; by the subcutaneous route, absorption is complete [4]. The low clearance and poor aqueous solubility constitute the pharmacokinetic basis for the six-monthly regimen [8,9]. No clinically relevant differences were identified as a function of age, sex, ethnic origin or body weight [5]. The drug is metabolised only to a limited extent and is eliminated essentially by the intestinal route, with the renal route playing only a secondary part [8,10].

Antiviral activity against HIV-1 is in the picomolar range, with mean EC50 values of 32 pM in CD4-positive T lymphocytes and 56 pM in macrophages, and approximately 50 pM across a panel of 23 HIV-1 isolates [11]. By contrast, there is a greater than tenfold reduction in susceptibility of HIV-2 [12]. This limitation is also suggested, anecdotally, by a very small case series: in the French ANRS-MIE CO5 cohort, only one of eight patients with multidrug-resistant HIV-2 infection achieved virological suppression despite six months of therapy [13].

The capsid is formed by the association of 200-250 hexamers and 12 pentamers in a fullerene cone configuration [14,15]. In this process, inositol hexakisphosphate plays a determining role [16]. Lenacapavir binds to the hydrophobic phenylalanine-glycine pocket formed by two adjacent subunits and stabilises the intra- and inter-hexameric interactions through a network of hydrogen bonds involving residues N57, K70, N74, S41, Q179, and N183 [17]. This hyperstabilisation prevents the controlled disassembly of the capsid required for reverse transcription and integration [17]. At the same time, by occupying the pocket to which cellular cofactors such as CPSF6 and NUP153 bind, it exerts competitive antagonism and prevents nuclear entry of the proviral DNA [17]. In the late phase, it diverts the assembly pathway by favouring stabilisation of the hexamers, preventing the number of pentamers needed for capsid closure from being reached and leading to the release of particles incapable of establishing infection [18]. The location of the resistance mutations in the residues of this hydrogen-bond network follows directly from this mechanism, a point to which we return below.

Clinical efficacy in treatment

The trials conducted to date span three distinct populations; the key data are summarised in Table 1.

**Table 1:** Clinical trials of lenacapavir in the treatment of HIV-1 infection BIC: bictegravir; FTC: emtricitabine; ISL: islatravir; LEN: lenacapavir; SC: subcutaneous; TAF: tenofovir alafenamide; W: week. Comparators: BIC/FTC/TAF in CALIBRATE, ARTISTRY-2, ISL+LEN, and ISLEND-1; the prior complex regimen in ARTISTRY-1; standard oral therapy in ISLEND-2. CAPELLA has no comparator arm. In the primary outcomes, the paired values refer, respectively, to the lenacapavir regimen and to the control group. For the phase 3 switch trials (ARTISTRY-1, ARTISTRY-2, ISLEND-1, and ISLEND-2), the primary endpoint was the proportion with HIV-1 RNA ≥50 copies/mL at week 48 (FDA snapshot algorithm), evaluated against a prespecified non-inferiority margin of 4 percentage points. The treatment difference (lenacapavir regimen minus comparator) and its 95% CI are expressed in percentage points. CALIBRATE and the phase 2 ISL+LEN study are reported descriptively, with no formal non-inferiority test; in CAPELLA, suppression is reported with missing data excluded.

Study (phase)	Population	n	Lenacapavir regimen	Primary outcome
CALIBRATE (2) [19]	Treatment-naive	182	LEN 927 mg SC every 26 weeks + FTC/TAF (200/25 mg) oral daily; at week 28, continuation with TAF, or BIC, or LEN/FTC/TAF (50/200/25 mg) oral daily	W54; HIV-1 RNA <50 copies/mL 85–90% (continuation groups) vs 92%
ARTISTRY-1 (3) [20]	Suppressed on a complex regimen	557	BIC/LEN (75/50 mg) oral daily	W48; HIV-1 RNA ≥50 copies/mL 1% vs 1%; difference −0.3 (95% CI −2.3 to 1.8); within the non-inferiority margin; no emergent resistance
ARTISTRY-2 (3) [21]	Suppressed on BIC/FTC/TAF	574	BIC/LEN (75/50 mg) oral daily	W48; HIV-1 RNA ≥50 copies/mL 1.3% vs 1.0%; difference 0.3 (95% CI −1.9 to 2.4); within the non-inferiority margin
ISL + LEN (2) [22]	Suppressed on BIC/FTC/TAF	104	ISL/LEN (2/300 mg) oral weekly	W48; HIV-1 RNA <50 copies/mL 94.2% vs 92.3%
ISLEND-1 (3) [23]	Suppressed on BIC/FTC/TAF	607	ISL/LEN (2/300 mg) oral weekly	W48; HIV-1 RNA ≥50 copies/mL 0% vs 0.3%; difference −0.3 (95% CI −1.4 to 0.8); within the non-inferiority margin
ISLEND-2 (3) [24]	Suppressed on standard therapy	626	ISL/LEN (2/300 mg) oral weekly	W48; HIV-1 RNA ≥50 copies/mL 0.3% vs 1.3%; difference −1.0 (95% CI −3.0 to 1.1); within the non-inferiority margin
CAPELLA (2/3) [25,26]	Multidrug-resistant infection	72	LEN 927 mg SC every 26 weeks + optimised background regimen	W156; HIV-1 RNA <50 copies/mL 85% (observed, missing data excluded); CD4 +164 cells/µL

In treatment-naive individuals, the phase 2 CALIBRATE study described rates of virological suppression at week 54 of between 85% and 90% in the lenacapavir arms and of 92% in the control arm, with wide and overlapping confidence intervals [19]. The data are compatible with efficacy similar to that of the standard daily oral regimen, but the level of evidence in this population did not go beyond phase 2.

It is in the planned regimen switch that the evidence is most robust. In these phase 3 switch trials, the primary endpoint was the proportion with HIV-1 RNA ≥ 50 copies/mL at week 48 (FDA snapshot algorithm), evaluated against a prespecified non-inferiority margin of 4 percentage points. For the fixed-dose combination of bictegravir/lenacapavir, the difference from the comparator lay within this margin both in patients on a complex regimen (ARTISTRY-1) and in those maintaining a single-tablet regimen (ARTISTRY-2), with no development of resistance observed in the former [20,21]. The same was observed for the weekly oral combination of islatravir/lenacapavir compared with standard therapy in the ISLEND-1 and ISLEND-2 studies [23,24]. The between-group difference and its 95% CI for each trial are shown in Table 1. These results demonstrate that the concept of long-acting therapy can be realised not only by the injectable route but also through less frequent oral regimens. Islatravir's development had earlier been placed on FDA clinical hold (December 2021) because of dose-related declines in CD4+ T-cell and total lymphocyte counts at the higher doses then under study [27,28]. The decisive finding that made this latter approach feasible was therefore the absence of this lymphocyte reduction at the low 2 mg weekly dose of islatravir [22]. None of these combinations has yet been authorised.

The CAPELLA study enrolled adults and adolescents with extensive treatment experience, with resistance to at least two drugs in each of at least three of the four main antiretroviral classes and with plasma HIV-1 RNA ≥ 400 copies/mL at screening, on a failing regimen [25,29]. Here, a methodological distinction assumes critical importance. By the Food and Drug Administration snapshot algorithm, the proportion with HIV-1 RNA < 50 copies/mL at week 156 was 61%, which suggested a considerable loss of efficacy. However, of the 27 participants without suppression, only 10 had genuinely detectable viraemia; the remainder corresponded to discontinuations or missing data. Excluding the missing data, the proportion with < 50 copies/mL at week 156 was 85% (44/52), and the mean increase in CD4 count reached 164 cells/µL [26]. This difference shows that, in long-term follow-up, the snapshot analysis reflects not only therapeutic success but also the effect of discontinuations and missing data.

Clinical efficacy in prevention

The findings in the domain of prophylaxis lie on a qualitatively distinct plane (Table 2).

**Table 2:** The PURPOSE programme, lenacapavir in pre-exposure prophylaxis FTC/TAF: emtricitabine-tenofovir alafenamide; FTC/TDF: emtricitabine-tenofovir disoproxil fumarate; LEN: lenacapavir; PrEP: pre-exposure prophylaxis; IRR: incidence rate ratio; background: background HIV incidence; PURPOSE: Preventing HIV with Underrepresented Populations through Engagement Incidences are presented per 100 person-years, and incidence rate ratios are shown with 95% CIs. In PURPOSE 1, the FTC/TAF arm did not differ significantly from the background incidence.

Study (phase)	Population	n	Comparator	Efficacy outcome (incidence per 100 person-years)
PURPOSE 1 (3) [30]	Adolescent girls and young women, 16-25 years; South Africa, Uganda	5338	FTC/TAF, FTC/TDF, background incidence	LEN 0 (0/2134); FTC/TAF 2.02; FTC/TDF 1.69; background 2.41. LEN vs background IRR 0.00 (95% CI 0.00 to 0.04); FTC/TAF vs background IRR 0.84 (95% CI 0.55 to 1.28)
PURPOSE 2 (3) [31]	Cisgender men, transgender and gender-diverse people, ≥ 16 years; seven countries	3265	FTC/TDF, background incidence	LEN 0.10 (2/2183); FTC/TDF 0.93; background 2.37. LEN vs background IRR 0.04 (95% CI 0.01 to 0.18); LEN vs FTC/TDF IRR 0.11 (95% CI 0.02 to 0.51)
PURPOSE 1 pregnancy substudy [32]	Participants who became pregnant	487	FTC/TAF, FTC/TDF	No infection with LEN; 5 infections in the comparator arms
PURPOSE 3 (2) [33]	Cisgender women; USA	253	FTC/TDF	Ongoing: pharmacokinetics, safety, acceptability
PURPOSE 4 (2) [34]	People who inject drugs; USA	181	FTC/TDF	Ongoing: pharmacokinetics, safety, acceptability
PURPOSE 5 (2) [35]	Adults; Europe	-	FTC/TDF	Ongoing: continuation of use
PURPOSE 365 (3) [36]	People with an indication for PrEP, ≥ 16 years	-	-	Ongoing: annual intramuscular administration, single arm

In the Preventing HIV with Underrepresented Populations through Engagement (PURPOSE 1) study, conducted in adolescent girls and young women in South Africa and Uganda, no HIV-1 infection was recorded among the 2,134 participants in the lenacapavir arm; across the study as a whole, over 4,821 person-years of follow-up, 55 infections were identified [30]. Since a placebo arm would not have been ethically acceptable, the background incidence estimated from the screened population was used as the comparator, a design regarded as a methodological innovation in PrEP research.

In the same study, the incidence in the F/TAF arm did not differ significantly from the background incidence (incidence rate ratio (IRR): 0.84, 95% CI: 0.55-1.28; p = 0.21). This absence of protection reflects poor adherence rather than a lack of pharmacological efficacy: among the preselected 10% of participants who underwent drug-level testing, most in both oral arms had tenofovir-diphosphate concentrations consistent with low adherence, which declined over time; 34 of 37 incident infections in the F/TAF group occurred in participants with low or undetectable concentrations; and medium-to-high adherence was associated with markedly lower odds of HIV acquisition (odds ratio: 0.11, 95% CI: 0.01-0.49) [30]. By contrast, lenacapavir reduced HIV incidence by essentially 100% relative to the background incidence (IRR: 0.00, 95% CI: 0.00-0.04; p < 0.001) and to F/TDF (IRR: 0.00, 95% CI: 0.00-0.10; p < 0.001), a superiority attributable in substantial part to the six-monthly regimen removing the need for daily adherence. In the PURPOSE 2 study, the oral regimen of the same class did confer protection in its population relative to the background incidence (FTC/TDF: 0.93 vs. 2.37), with lenacapavir reducing HIV incidence by 96% relative to the background incidence (IRR: 0.04, 95% CI: 0.01-0.18) and by 89% relative to F/TDF (IRR: 0.11, 95% CI: 0.02-0.51) [31]. The difference between the two studies lies not in the drug itself but in the therapeutic adherence of the populations. In PURPOSE 2, in the two participants who acquired infection in the lenacapavir arm, despite plasma concentrations within the expected range, the N74D capsid mutation was detected at the time of diagnosis [37]. The significance of this finding is addressed in the section on resistance.

The pregnancy substudy of PURPOSE 1 deserves particular appraisal. It is the first phase 3 PrEP trial in which participants who became pregnant were able to continue the study drug. Among the 193 pregnancies in the lenacapavir group (184 participants), no HIV acquisitions occurred during pregnancy, and no vertical transmissions were observed. Pregnancy outcomes were broadly consistent with background values: stillbirth in five of 195 outcomes (3%; regional range: 2.0-4.0%) and spontaneous abortion in 20 of 195 (10%; global range: 10-20%). Infant drug exposure through breast milk was minimal (breastfed-infant-to-maternal plasma ratio: 0.02), and congenital anomalies were reported in six of 127 infants (5%), numerically higher than in the F/TAF group (3%) but considered by the investigators to be consistent with background incidence. Given the small absolute numbers, these findings are reassuring but, as the investigators caution, should not be interpreted as confirmatory of safety [32]. The molecule is detected in breast milk, but plasma exposure in breastfed infants remains minimal, which is consistent with the low oral bioavailability [32]. Given the need for safe and effective HIV prevention options in pregnancy, the inclusion of the assessment of pregnancy, breastfeeding and infant pharmacokinetics within a large, adequately controlled PrEP trial constitutes a methodological gain. By contrast, therapeutic use in pregnant women with HIV-1 infection is documented only anecdotally, in a single case report in which N74D resistance emerged five weeks after injection, at 32 weeks of gestation [38].

Resistance and the pharmacokinetic tension

The most critical tension brought out in this review lies in the fact that the pharmacokinetic property that gives lenacapavir its strength constitutes at the same time its principal vulnerability. To assess this tension, it is necessary first to characterise the molecular basis of resistance.

The associated mutations are located in the residues of the binding pocket and are classified in the 2025 IAS-USA update as major (L56I, M66I, Q67H, K70H/N, N74D) and minor (K70S/R, N74S, A105T, T107N) (Table 3) [39].

**Table 3:** Resistance mutations to lenacapavir FG: phenylalanine-glycine

Mutation	Classification	Structural basis	Clinical observation
M66I	Major	Steric hindrance by the β-branched side chain; does not affect the binding of cofactors with FG motifs	Most frequent mutation at week 26 in CAPELLA (6/8); highest level of resistance
Q67H	Major	The closed conformation of His67 obstructs the pocket; requires a conformational transition for drug binding	Selected chiefly at concentrations < 5 ng/mL
N74D	Major	Loss of the direct hydrogen bond and electrostatic repulsion; about 20-fold reduction in affinity	Selection window overlaps the steady state of the six-monthly regimen
L56I, K70H/N	Major	Residues of the binding pocket; structural basis not characterised in detail	Identified in clinical trials
K70S/R, N74S, A105T, T107N	Minor	Residues of the binding pocket; structural basis not characterised in detail	Limited impact in isolation

Q67H acts by steric hindrance: in the unbound protein, the side chain of His67 projects into the interior of the pocket in a closed conformation and must pass to the open conformation to accommodate lenacapavir; it is proposed that this transition explains the reduced potency of the inhibitor [39]. N74D leads to loss of the direct hydrogen bond and to the emergence of electrostatic repulsion, with a reduction of about 20-fold in binding affinity [39]. M66I confers the highest level of resistance through a specific steric hindrance that does not affect the binding of cofactors bearing phenylalanine-glycine motifs [40,41]. Since these mutations rarely occur as natural polymorphisms, genotyping of the capsid before initiating lenacapavir has limited clinical utility [42].

The origin of the vulnerability lies less in the molecule than in its exposure profile. The prolonged half-life leaves, after discontinuation, a tail of progressively decreasing concentrations: in an average individual, the concentration only falls below 5 ng/mL about 11.8 months after the last 927 mg injection, a period in which the preferential selection of Q67H has been described [43]. Exposure to the virus within this window creates a context of monotherapy. Modelling studies point to a risk of de novo resistance developing between 106 and 235 days after the injections are stopped, depending on the type of mutation, which demonstrates that the problem is not merely theoretical [43]. The concentrations that confer complete protection vary between 21 ng/mL for Q67H and 1142 ng/mL for the triple mutant Q67H+K70R+T107N [43]. These protective concentrations (the levels required to fully suppress each mutant) are distinct from the concentrations at which the mutants are selected: Q67H, for instance, is selected chiefly below 5 ng/mL, whereas its complete suppression requires approximately 21 ng/mL.

The most relevant finding of this same work is, however, a different one: a mutant selection window is the concentration range in which the wild-type virus is suppressed but a resistant mutant can still be selected; for N74D and for all of the double mutants, these windows overlap the steady-state concentrations observed with six-monthly subcutaneous administration [43]. The clinical correlate of this prediction was observed in the prevention studies: in PURPOSE 2, in the two participants who acquired infection in the lenacapavir arm, despite plasma concentrations within the expected range, it was precisely N74D that was detected at the time of diagnosis, that is, the mutation predicted by the modelling [37]. The risk of selection therefore exists not only in the period of decline following discontinuation but is also present during regular maintenance of the regimen.

In CAPELLA, in turn, in all 14 participants who developed resistance, either the background regimen did not include any fully active drug (n = 4) or the plasma concentrations of those drugs were insufficient (n = 10) [26,44]. In seven of these participants, viraemia was again suppressed after adherence to the background regimen was resumed or fully active agents were introduced [44].

Resistance in treatment therefore appears to be more closely associated with the conditions of use than with the molecule itself; the vulnerability is manageable, but this depends on therapeutic adherence and on combination with at least one or two fully active antiretrovirals [44].

This assessment is not transferable to prevention: in PrEP, lenacapavir is, by definition, a monotherapy. This does not constitute an argument against its use in prevention; in the PURPOSE studies, resistance also emerged in the oral comparator arms, where four seroconverters developed resistance (two of 37 tested in the F/TAF arm and one of 16 in the F/TDF arm in PURPOSE 1, and one of nine in the F/TDF arm in PURPOSE 2), consisting of emtricitabine resistance (M184I/V) in all four and tenofovir resistance (K65R) in one [37].

Rather, it indicates that the risk must be managed by another route: it is argued that the decisive step is not the companion drug but the reliable exclusion of infection before initiation and throughout use, together with rapid transition to a fully active regimen once a diagnosis is established. In practice, three measures follow from this. Screening before initiation and before each injection should use an assay able to detect acute infection, and a negative antigen/antibody result should be confirmed by an HIV-1 nucleic acid (RNA) test, since antibody-based assays alone may be insufficient in this setting [45]. The need for sensitive virological testing is reinforced by experience with long-acting cabotegravir, where breakthrough infection can present with low or undetectable viraemia and delayed or blunted seroconversion (long-acting early viral inhibition), delaying antibody-based diagnosis and favouring resistance; whether this pattern also occurs with lenacapavir is not yet established [46]. Finally, because residual lenacapavir concentrations can persist for up to 12 months or longer, individuals who discontinue and remain at risk should begin an alternative, usually oral, PrEP regimen within 28 weeks of the last injection to cover this pharmacokinetic tail [45].

In the event of virological failure, resistance monitoring requires genotypic assessments that include the region of the capsid gene [47].

Structural knowledge of these mechanisms likewise opens the way to second-generation capsid inhibitors with a higher barrier to resistance, such as the resistance-tolerant 4-quinazolinone-containing compounds in preclinical development [40,48], and clinical-stage agents such as VH499 (VH4011499), which showed antiviral activity in a phase 2a proof-of-concept trial [49]. 

Safety and tolerability

With respect to the safety of lenacapavir itself, the most common adverse reactions are injection-site reactions. In the CAPELLA study, these occurred in about 65% of participants (predominantly swelling in 36%, pain in 31%, erythema in 31%, nodules in 25%, and induration in 15%) and were mostly grade 1 or 2; subcutaneous nodules and induration were the slowest to resolve. Nausea was reported in about 4% of participants. The drug was otherwise well tolerated, with only around 1% of participants discontinuing because of adverse events [5].

Safety in pregnancy and lactation, including pregnancy outcomes and infant exposure through breast milk, is addressed in the prevention section (PURPOSE 1 pregnancy substudy), and the use of lenacapavir in hepatic or renal impairment and in adolescents is considered below.

Implications for clinical practice

The interaction potential of lenacapavir is globally limited: it is a moderate inhibitor of cytochrome P450 3A (CYP3A) and a weak inhibitor of P-glycoprotein (P-gp) [50]. However, the prolonged half-life requires consideration of an interaction window that extends to as much as nine months after the last subcutaneous dose (Table 4) [5].

**Table 4:** Clinically relevant drug interactions CYP3A: cytochrome P450 3A; CYP3A4: cytochrome P450 3A4; P-gp: P-glycoprotein; UGT1A1: uridine diphosphate glucuronosyltransferase 1A1

Situation	Effect	Management
Potent CYP3A inducers (rifampicin, carbamazepine, phenytoin, St John's wort)	Rifampicin reduces the area under the curve of lenacapavir by 84%	Contraindicated
Moderate CYP3A inducers (e.g. efavirenz)	56% reduction in the area under the curve of lenacapavir	Not recommended
Simultaneous inhibition of P-gp, UGT1A1 and CYP3A (atazanavir/cobicistat)	4.21-fold increase in exposure	Not recommended
Darunavir/cobicistat	94% increase in the area under the curve of lenacapavir	No dose adjustment
Clopidogrel	Reduced conversion to the active metabolite by CYP3A4	Consider an alternative antiplatelet agent
Narrow-therapeutic-index antiarrhythmics (amiodarone, disopyramide, quinidine)	Increased exposure to the antiarrhythmic	Not recommended
Calcium-channel blockers metabolised by CYP3A4	Increased exposure to the blocker	Monitor blood pressure; adjust the dose
Antineoplastics metabolised by CYP3A4, chiefly kinase inhibitors	Risk of antineoplastic toxicity	Consult a drug-interaction database

The consequence with the greatest global reach is the contraindication of concomitant use with potent CYP3A inducers, among them rifampicin, which reduces the area under the curve of lenacapavir by 84% [5]. Given the burden of tuberculosis in people living with HIV, especially in the regions of highest incidence, this limitation constitutes a relevant practical obstacle. Many antineoplastics, chiefly the kinase inhibitors, which play a growing role in oncological treatment, are metabolised by CYP3A4; given their toxicity profiles, caution is recommended in concomitant use with lenacapavir [51].

In special populations, phase 1 data support use without dose adjustment in mild to moderate hepatic impairment (Child-Pugh A and B) and in mild, moderate, or severe renal impairment (creatinine clearance: 60-89, 30-59, and 15-29 mL/min, respectively, by Cockcroft-Gault); the pharmacokinetics have not been studied in severe hepatic impairment [52].

There are no data on use below 16 years of age; in adolescents aged 16 to under 18 years and weighing 35 kg or more, safety was assessed in 59 participants of the PURPOSE 1 and 2 studies, with adverse reactions consistent with those observed in adults [5].

As regards the combinations, the level of evidence is heterogeneous. Beyond the phase 3 combinations already mentioned, the association with cabotegravir is at the level of retrospective case series: in a series of 34 patients who switched regimen because of adherence difficulty or underlying resistance, the proportion with virological suppression rose from 47% to 94% over a median period of eight weeks [53]. The association with broadly neutralising antibodies remains at the proof-of-concept level, limited by the need for prior determination of susceptibility and by the intravenous route [54].

Access and implementation

The translation of this efficacy into public-health benefit will depend heavily on affordability and delivery. In high-income settings, the list price of the PrEP formulation is high (around US$28,000 per person per year) [55], but Gilead has signed voluntary licensing agreements enabling generic manufacturers to supply the drug [56], with generic versions anticipated at approximately US$40 per person per year in low- and middle-income countries [55]. Agreements with the Global Fund to Fight AIDS, Tuberculosis and Malaria (the Global Fund) [57] and the US President’s Emergency Plan for AIDS Relief (PEPFAR) [58] aim to provide lenacapavir at no profit to up to two million people, primarily in low- and lower-middle-income countries, and in July 2025 the World Health Organization recommended twice-yearly injectable lenacapavir as an additional PrEP option [59]. Real-world implementation will nonetheless hinge on delivery infrastructure, regular HIV testing, and equitable scale-up.

## Conclusions

As the first drug directed at the HIV-1 capsid, lenacapavir represents one of the most significant pharmacological and therapeutic innovations of recent years in antiretroviral care, making long-acting administration a feasible option in both treatment and prevention. Nevertheless, the limited genetic barrier to the development of resistance (single capsid substitutions, such as M66I, Q67H, and N74D, can reduce susceptibility) and the prolonged pharmacokinetic tail demonstrate that the very characteristics that make it effective constitute at the same time its most fragile facet. The fact that resistance is associated with the conditions of use suggests that this vulnerability is manageable; the fact that the risk is not confined to the period of discontinuation but is also present during maintenance demands particular attention.

Lenacapavir should therefore not be understood as a standalone solution, but as an instrument that acquires its full meaning only when combined with careful patient selection and monitoring, with at least one or two fully active drugs, with rigorous adherence, and with a planned discontinuation strategy. Closing the data gap in pregnant women and in the paediatric population, developing alternative inhibitors, and reducing inequalities in access are prerequisites for this potential to translate into public health gains.
